# Development of a Distinct Microbial Community Upon First Season Crop Change in Soils of Long-Term Managed Maize and Rice Fields

**DOI:** 10.3389/fmicb.2020.588198

**Published:** 2020-11-09

**Authors:** Katharina Frindte, Sarah A. Zoche, Claudia Knief

**Affiliations:** Institute of Crop Science and Resource Conservation – Molecular Biology of the Rhizosphere, University of Bonn, Bonn, Germany

**Keywords:** rice-maize crop rotation, paddy soil, upland soil, continuous cropping system, flodding of soils, bacterial and fungal communities, rhizosphere microbiota, cropping history

## Abstract

The introduction of crop rotation regimes in paddy soils, for example, rice in combination with maize, implements the establishment of new paddy fields to compensate for reduced rice production on existing fields. To study responses of the soil and rhizosphere microbiota upon introduction of a new crop species into continuous cropping agroecosystems, we conducted experiments with soils from adjacent fields where rice and maize were grown successively for more than 30 years. In microcosm experiments, rice and maize plants were cultivated in both soils under the respective plant-required management regime, i.e., rice cultivation under flooded conditions and maize under non-flooded conditions. 16S rRNA gene and fungal ITS region amplicon analysis showed that the soil and rhizosphere microbiota was clearly distinct between soils after long-term rice/maize management. Upon change of the management regime, the bulk soil microbiota became different to both, the former microbial community in the soil and the community being characteristic for the respective type of long-term cropping. Nevertheless, the influence of the soil management history remained clearly visible besides the impact of the new management regime. Similar results were observed for the rhizosphere, though the combined effect of plant species and altered management was even more effective in this compartment compared to the bulk soil. The newly introduced crop plant did not take over characteristic members of the rhizosphere microbiota of the previously cultivated crop; instead, some previously rare taxa became enriched. Thus, the formerly grown crop species did not directly affect the recruitment of microorganisms in the rhizosphere of the following crop species. Further, the results show that the rhizosphere and bulk soil microbiota do not develop straight toward the specific microbiota that is characteristic for a continuous cropping system, but reach a distinct stage upon introduction of a new crop species and new management practices.

## Introduction

Rice and maize represent besides wheat the most important staple foods worldwide. The demand for more food for a growing human population is met by increasing crop yield, harvesting frequency, and cropland area [Ray et al., 2012; Food and Agriculture Organization of the United Nations (FAOSTAT)]. The cultivation of maize has further been promoted due to increased biofuel and meat production (Rosegrant, 2008). An attractive option to fulfill the increasing demand for these crops is the introduction of maize in paddy soils by converting rice-rice to rice-maize cropping systems in Southeast Asia (Timsina et al., 2010). This reduces the problem of limited irrigation water availability during the dry season when maize is grown (Tuong et al., 2005) and reduces the carbon footprint (Jiang et al., 2020). However, the conversion of continuous rice cropping systems into rice-maize crop rotations leads to a lower production of rice per acre per year. Moreover, rice paddies are lost, e.g., in the Southeast of Asia, due to other reasons such as urbanization (Zhang et al., 2017). Thus, rice production has to be increased in other regions, e.g., by establishing new rice paddies or introducing rice-rice double or even triple cropping instead of single cropping. A remarkable expansion of rice paddies by conversion of croplands or natural wetlands has been observed during the last 15 years in the northeast of China and in Northern India (Dong et al., 2016; Zhang et al., 2017). Besides, the conversion of croplands into paddy soils and vice versa occurs regularly in other rice cropping regions, e.g., in the southeast of China (Jiang et al., 2019).

Conversions between upland and paddy soils lead to very different physical, chemical, and biological conditions in the soils, because paddy soils promote the development of anoxic conditions upon flooding (Timsina and Connor, 2001). This causes substantial changes in the soil microbiota. Such changes have been seen in paddy soil chronosequence studies (Cui et al., 2012; Liu et al., 2016b; Ding et al., 2017), but also in paddy soils that underwent different crop rotation regimes in combination with rice (Xuan et al., 2012; Lopes et al., 2014; Zhao et al., 2014; Breidenbach and Conrad, 2015; Maarastawi et al., 2018). The reduced flooding upon change from continuous rice cropping toward rice maize crop rotation leads to microbial communities that remain similar to those of paddy soils with diverse facultative and obligate anaerobic bacteria. Differences in microbial community composition in dependence on crop rotation result from the different management practices, including primarily the water management and linked to it soil aeration (Maarastawi et al., 2018), but are also dependent on the cultivated crop, the different fertilizer applications, plant residue input, tillage or pest control treatments (Thiele-Bruhn et al., 2012). Apart from abiotic soil properties that are altered due to changes in field management, the cultivated plants induce changes by enriching specific microorganisms in the rhizosphere (Peiffer et al., 2013; Edwards et al., 2015; Hu et al., 2018), which remain to some extent in the soil even after harvest.

The rhizosphere is a specific microbial hot spot region in the soil, where microorganisms are primarily promoted by plant root released carbon (Bais et al., 2006; Badri and Vivanco, 2009). Besides, the microorganisms in the rhizosphere are affected by diverse other biotic and abiotic factors, similarly as microorganisms in the bulk soil. It is for example, well-known that the rhizosphere microbiota of plant species such as rice or maize differ in dependence on soil type and are affected by cultivation practices (Peiffer et al., 2013; Edwards et al., 2015; Maarastawi et al., 2018). Moreover, the cropping history of a field affects microbial communities in the rhizosphere (Benitez et al., 2017; Hansen et al., 2018; Li et al., 2019). Effects of long-term continuous cropping on soil microbial communities have been addressed in different studies (e.g., Cui et al., 2012; Liu et al., 2016a; Ding et al., 2017; Li et al., 2017; Babin et al., 2019), but the immediate changes occurring in the soil and rhizosphere microbiota upon a modification in crop cultivation have gained less attention, even though this transition phase may exert specific effects on plant performance and the associated microbiota.

In this study, we analyzed differences in rhizosphere and soil microbial communities that result from long-term continuous cropping vs. crop change from rice to maize cultivation and vice versa. We performed microcosm experiments with a complementary design by converting a long-term paddy soil (rice soil, RS) into an upland soil used for maize cultivation and by converting an upland soil used for long-term maize cultivation (maize soil, MS) into a paddy soil. For comparison, maize plants were additionally grown in the long-term MS and rice plants in the long-term RS. The soils were taken from adjacently located agricultural fields that were managed under maize and rice cultivation for >30 years, respectively. The experiment with maize plants and the corresponding sequencing data were part of a larger study by Maarastawi et al. (2018), while the setup with rice plants was performed specifically for this study. The focus of the previous study was on microbial responses when converting rice-rice into rice-maize cropping systems and the longer-term effects of this specific type of crop rotational regime on the soil microbiota. In this study, we aimed to disentangle the effects of cropping history and cultivated crop with its accompanied management regime on rhizosphere and bulk soil microbial communities specifically in the first season upon rice-maize and maize-rice crop change after long-term continuous cropping. The study design allowed us to evaluate effects due to crop rotational management very specifically, as the long-term continuous cropping has left detectable traces in the soil microbiota (Maarastawi et al., 2018), which we aimed to retrieve in the opposite type of soil microbiota upon crop rotational change. We hypothesized (1) that land use changes affect microbial diversity in the bulk soil. We predict an increase especially in bacterial though less in fungal diversity upon first-time flooding of an upland bulk soil. In contrast, a decline in diversity is predicted for the RS microbiota when the soil is used for maize cropping under non-flooded conditions. This hypothesis is derived from the fact that RS hosts a higher microbial diversity than MS (Maarastawi et al., 2018) and the assumption that a transition toward the other type of soil microbiome occurs upon crop change, (2) a partial transition of the bulk soil microbial community composition is expected upon first-time management change with a development toward the community seen in the corresponding long-term managed bulk soil. We expect stronger changes in MS that is flooded for the first-time than for the non-flooded RS, (3) for the rhizosphere microbiota, we hypothesize that the newly introduced plant species will partially take over the rhizosphere microbiota of the previously grown crop, as a well-adapted host-plant specific microbiota is not yet established in a soil in which this plant species has not been cultivated before. Instead, the previously grown crop has left its footprint in the soil microbiome and will influence colonization of the rhizosphere. (4) Moreover, we expect to see an increase in alpha diversity in the rhizosphere if a crop plant is grown in a soil in which it has not been cultivated before. We explain this by a less specific recruitment of microorganisms from the bulk soil, as the microbiota in that soil is not yet highly adapted to the new crop species.

## Materials and Methods

### Sampling Sites

Fields for soil sample collection were located in Zeme, Italy (45°11.536′N, 8°40.078′E). The soils were taken from two neighboring fields, one undergoing continuous rice cropping for >30 years (RS) and the other one with maize for >30 years (MS). Information about the soil properties is published in Maarastawi et al. (2018). The soils were collected in spring 2015 before rice/maize planting and after fertilization with 100 kg ha^−1^ horn and hoof. The soils were transferred to the lab, immediately air dried, well mixed and stored at room temperature before they were used for the microcosm experiments.

### Setup of the Microcosm Experiments

Microcosm experiments with maize (variety NC358) and rice (variety Nipponbare) were conducted in greenhouses at the University of Bonn in 2015 and 2017, respectively. For maize plants, 7-L plastic pots were filled with MS or RS, while 5-L buckets were used for rice plants. Maize seeds were immediately sown in the soil, while rice plants were grown for 2 weeks in hydroponic pre-culture before transplanting (see Ueda et al., 2016). Maize plants were watered daily, while rice plants were grown in constantly flooded soils. For bulk soil analyses, unplanted pots were prepared. The experimental setup included four replicates for maize microcosm experiments and three for rice. NPK fertilization was performed according to the needs of the respective plants (details in Ueda et al., 2016; Maarastawi et al., 2018).

### Rhizosphere and Bulk Soil Sample Collection

Bulk soil and rhizosphere samples were taken after 3 months of plant growth. Bulk soil samples were additionally taken at the day of transplanting/sawing (day 0). The soil used for rice cultivation was flooded 24 h before planting and sampling. Bulk soil samples were taken from non-flooded soils in each pot with a spatula, while bulk soil samples of the flooded soil were taken from the upper 2 cm with a pipette after gentle mixing to include material from the oxic as well as the anoxic layer of the soil, as both contribute to the microbial processes in the bulk soil. Water was removed from flooded soil samples by centrifugation. Rhizosphere sampling was done using specifically adapted washing protocols for the two plant species as described earlier (Ueda et al., 2016; Maarastawi et al., 2018). At the end of the washing procedure, the obtained pelleted rhizosphere samples were resuspended in 1 ml TE buffer. All soil samples were stored at −20°C until further processing.

### Nucleic Acid Extraction and Amplicon Sequencing

Soil DNA extraction was performed using the NucleoSpin Soil Kit (Macherey Nagel, Düren, Germany) following the manufacturer’s instructions with few modifications as described in Maarastawi et al. (2018). 16S rRNA genes were amplified using barcoded 515F primers in combination with the 806R primer (Frindte et al., 2019), which target the 16S rRNA gene of *Bacteria* and *Archaea* (Bates et al., 2011). Throughout the manuscript, we refer in this context to bacteria, having in mind that both domains are covered. The fungal ITS1 region was amplified using the primer set ITS1F-ITS2 (White et al., 1990; Gardes and Bruns, 1993). Detailed PCR protocols have been published in Maarastawi et al. (2018). The PCR products were quantified using the QuantiFluor dsDNA System (Promega, Madison, United States) on an Infinite 200 Pro plate reader (Tecan, Männedorf, Switzerland) and pooled at equimolar concentrations. Pooled PCR products were cleaned using the CleanPCR magnetic bead system (CleanNA; Alphen aan den Rijn, Netherlands) according to manufacturer’s instructions. Library preparation and sequencing on an Illumina HiSeq 2500 system was done by the Max Planck-Genome-centre Cologne and resulted in paired-end reads of 2 × 250 bp. Read files have been submitted to the ENA public database, entry PRJEB23682 includes the previously generated maize cultivation sequence dataset as part of the study of Maarastawi et al. (2018), PRJEB35017 contains the rice cultivation sequence dataset of this study.

### Bioinformatics and Statistics

Sequence processing including assembly of the paired end reads, quality control, and operational taxonomic unit (OTU) clustering using a 97% similarity threshold was performed as described in Maarastawi et al. (2018). The resulting OTU table was rarefied to obtain equal read numbers for all samples (bacteria: 11,794 reads, fungi: 1,751 reads).

Chao1 and Pilous’s evenness values were calculated in R using the package vegan to evaluate alpha-diversity (Oksanen et al., 2019; R Core Team, 2020). Overall differences between groups of samples, including all time points were assessed based on non-parametric Mann-Whitney tests, because data were non-normally distributed. Comparisons were made in dependence on cropping history (long-term cultivation of rice vs. maize), management/plant (flooded/rice vs. non-flooded/maize) or compartment (bulk soil vs. rhizosphere). For a more detailed analysis, data were separated according to compartment, cropping history, and management/cultivated crop plant and by considering only bulk soils sampled after 3 months. A normal distribution of these datasets and equal variances allowed us to apply ANOVA with Tukey HSD *post hoc* tests at a significance level of *p* < 0.05 in SPSS 21.0 (SPSS, Inc., United States).

Non-metric multidimensional scaling (NMDS) plots were built and Permutational Multivariate Analysis (ADONIS) calculated based on Bray-Curtis distance matrixes, which were derived from Hellinger transformed OTU tables (package “vegan” version 2.5-6 in R). ADONIS was performed to assess differences due to cropping history, effect of flooding and/or cultivated plant species, compartment, and time in all samples as well as to evaluate autocorrelations (AC) between these factors. These analyses were repeated for sub-datasets, which were separated by compartment, cropping history and/or management/cultivated plant species.

Responsive taxa being significantly enriched in relative abundance in specific groups of samples were identified at different taxonomic ranks using ANOVA with Tukey-Kramer *post hoc* analyses and a Benjamini-Hochberg false discovery rate correction in STAMP (Parks et al., 2014). Venn diagrams were set up using an online tool to illustrate counts of significantly enriched OTUs identified by STAMP.

## Results

### Changes in Bacterial and Fungal Alpha Diversity in Rhizosphere and Bulk Soil

Analysis of the effects of compartment, cropping history, management (incl. flooding) regime in combination with the cultivated plant and time (the latter for bulk soil data only) revealed that bacterial alpha diversity was more responsive to these factors than fungal diversity (Table 1). The strongest differences in estimated richness for both communities were observed in relation to cropping history. Likewise, the factor compartment affected diversity, but especially the bacterial diversity, not the fungal diversity. Changes in alpha diversity over time, analyzed at the beginning and end of the experiment in the bulk soil samples, were mostly not observed.

**Table 1:** Mean values and standard deviation (SD) of the Chao1 diversity index and Pilous’s evenness for samples grouped according to different factors.

Factor	Sample group	Chao1 bacteria	Evenness bacteria	Chao1 fungi	Evenness fungi
Compartment	Bulk soil	2,800 ± 657^**^	0.80 ± 0.058^*^	424 ± 157	0.66 ± 0.089
	Rhizosphere	2,234 ± 530	0.77 ± 0.043	349 ± 102	0.62 ± 0.072
Cropping history	Maize (MS)	2,127 ± 561^***^	0.77 ± 0.061^*^	292 ± 78^***^	0.64 ± 0.065
	Rice (RS)	3,096 ± 327	0.81 ± 0.039	506 ± 113	0.65 ± 0.100
Management (plant/flooding)	Maize/non-flooded	2,651 ± 651	0.81 ± 0.036^**^	394 ± 132	0.62 ± 0.065^**^
	Rice/flooded	2,560 ± 706	0.76 ± 0.060	406 ± 163	0.68 ± 0.093
Time (bulk soil only)	0 days	2,168 ± 446	0.78 ± 0.065^*^	424 ± 136	0.67 ± 0.076
	3 months	2,032 ± 586	0.82 ± 0.045	349 ± 130	0.65 ± 0.064

Mann-Whitney tests were used to detect significant differences between pairs of samples. Significance levels are indicated by asterisks in the respective upper line of each paired dataset. MS, maize soil; RS, rice soil.

* *p* < 0.05

** *p* < 0.01

*** *p* < 0.001.

**Figure 1:**
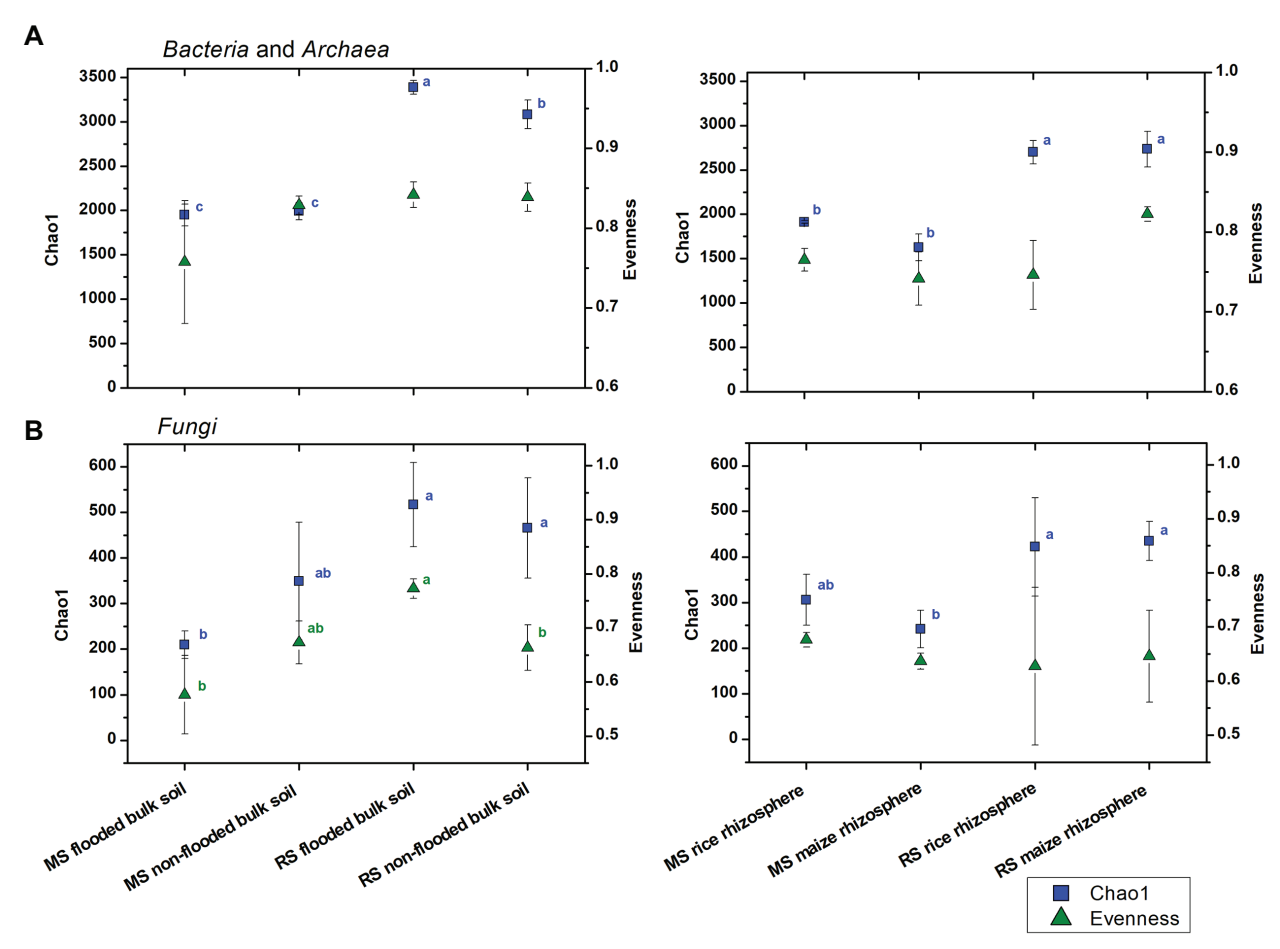
Changes in **(A)** bacterial and archaeal and **(B)** fungal alpha diversity measures in soils with different cropping history (maize soil, MS or rice soil, RS), management and cultivated crop plants. Mean values and standard deviations (SDs) of the diversity measures for bulk soil (left) and rhizosphere samples (right) are given. Significant differences between samples were assessed by ANOVA with Turkey HSD *post hoc* tests (*p* < 0.05) and are indicated by superscript letters in blue (Chao1) and green (evenness) when detected.

To evaluate the effects of cropping history and crop rotational change on alpha diversity more specifically, the bacterial and fungal datasets obtained after 3 months of incubation were used and separated by compartment (Figure 1). For the bulk soil samples, the overall trend of higher estimated richness in RS than MS was confirmed for bacteria and fungi (ANOVA, *p* < 0.001 and *p* = 0.005, respectively), while bacterial and fungal evenness was barely significantly affected by cropping history (*p* = 0.094 and *p* = 0.056, respectively). Strongest differences in estimated richness were observed between flooded RS, which had the highest bacterial and fungal richness indices (3,386 ± 78 and 517 ± 92, respectively), and flooded MS, which showed the lowest bacterial and fungal richness (1,948 ± 121 and 209 ± 30, respectively). The first-time flooding of MS did not significantly affect bacterial diversity and evenness in comparison to the non-flooded MS. Instead, fungal diversity and evenness tended to decrease, though not significantly (Figure 1). Non-flooding of RS left bacterial evenness and fungal diversity unaffected, but significantly reduced bacterial diversity and fungal evenness.

In the rhizosphere, cropping history affected bacterial and fungal richness as well (*p* < 0.001 and *p* < 0.001, respectively), with higher Chao1 diversity for plants grown in RS (bacteria: 2,717 ± 166, fungi: 429 ± 76) than in MS (bacteria: 1,770 ± 83, fungi: 274 ± 49). The two plant species hosted microbial communities of comparable richness when grown in soil of the same cropping history. This implemented that the rice rhizosphere bacterial communities declined in richness when grown in MS compared to RS, while richness increased in the maize rhizosphere bacterial communities when grown in RS compared to MS (Figure 1). Bacterial and fungal evenness did not show consistent responses to cropping history or cultivated plant species.

### Identification of Major Differences in Beta Diversity and Underlying Explanatory Factors

**Figure 2:**
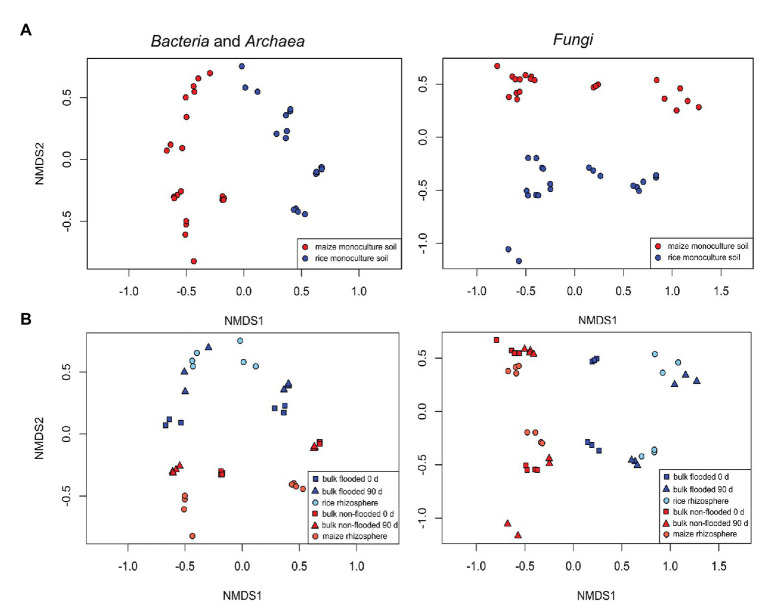
Non-metric multidimensional scaling (NMDS) plots showing differences in bacterial (left) and fungal (right) community composition due to **(A)** cropping history (upper panels) or **(B)** management/cultivated plant (lower panels, blue vs. red color). Additionally, compartment (light and dark color and symbol shape) and incubation period (symbol shape) are encoded in the lower panels. Flooded bulk soil was taken at day 0 was flooded 24 h before sampling.

A first comparative assessment of the effects of the grouping factors cropping history, management/cultivated plant species, compartment and incubation time on the bacterial and fungal community composition was made based on the full dataset. It showed that cropping history of the soil and the applied management along with the cultivated plant species was primarily associated with dissimilarities between samples. The bacterial community composition responded most strongly to cropping history (ADONIS *R* ^2^ = 0.24, *p* = 0.001; Table 2) followed by management (*R* ^2^ = 0.17, *p* = 0.001). In contrast, the fungal community showed a slightly stronger response to management (*R* ^2^ = 0.20, *p* = 0.001) than to cropping history (*R* ^2^ = 0.17, *p* = 0.001). These responses were clearly reflected in the NMDS plots, where samples were well separated along the first two axes according to these grouping factors (Figure 2). The differentiation of samples according to compartment was weak in the full dataset, though visible in the NMDS plots and confirmed based on ADONIS (bacteria: *R* ^2^ = 0.09, *p* = 0.001; fungi: *R* ^2^ = 0.05, *p* = 0.002). To evaluate effects of autocorrelation, factor combinations between cropping history, management, and compartment were included in ADONIS (Table 2). *R* ^2^ values were only partially significant and remained below 0.06 for all factor combinations, indicating that the three most relevant factors had primarily an independent impact on microbial community composition.

**Table 2:** Results of Permutational Multivariate Analysis (ADONIS) displaying the relevance of different factors that shape bacterial and fungal communities.

Data set representing dependent variable	Independent variables	Bacteria		Fungi	
		*R* ^2^	*p*	*R* ^2^	*p*
All data	Cropping history	0.24	0.001	0.17	0.001
	Management (flooding/plant)	0.17	0.001	0.20	0.001
	Compartment	0.09	0.001	0.05	0.011
	Time	0.04	0.001	0.03	0.001
	AC Cropping history + management	0.05	0.003	0.05	0.001
	AC Cropping history + compartment	0.03	0.060	0.02	0.344
	AC Management + compartment	0.05	0.015	0.04	0.013
Bulk soil	Cropping history	0.34	0.001	0.20	0.001
	Management (flooding)	0.18	0.001	0.19	0.001
	Time	0.06	0.001	0.05	0.006
	AC Cropping history + management	0.07	0.005	0.07	0.002
	AC Cropping history + time	0.03	0.019	0.03	0.032
	AC Management + time	0.06	0.001	0.06	0.003
Rhizosphere	Cropping history	0.23	0.001	0.19	0.003
	Management (flooding/plant)	0.37	0.001	0.39	0.001
	AC Cropping history + management	0.12	0.005	0.08	0.052
Bulk soil MS	Management (flooding)	0.37	0.001	0.35	0.001
	Time	0.15	0.003	0.08	0.021
	AC Flooding + time	0.18	0.001	0.14	0.006
Bulk soil RS	Management (flooding)	0.38	0.001	0.23	0.001
	Time	0.13	0.007	0.10	0.007
	AC Management + time	0.12	0.005	0.09	0.058
Flooded bulk soil	Cropping history	0.38	0.001	0.26	0.001
	Time	0.20	0.002	0.20	0.001
	AC Cropping history + time	0.09	0.071	0.13	0.002
Non-flooded bulk soil	Cropping history	0.58	0.001	0.38	0.001
	Time	0.12	0.008	0.08	0.049
	AC Cropping history + time	0.06	0.020	0.06	0.110

Autocorrelations (AC) between factors are included. Analyses were performed with the complete dataset and different sub-sets. MS, maize soil; RS, rice soil.

**Figure 3:**
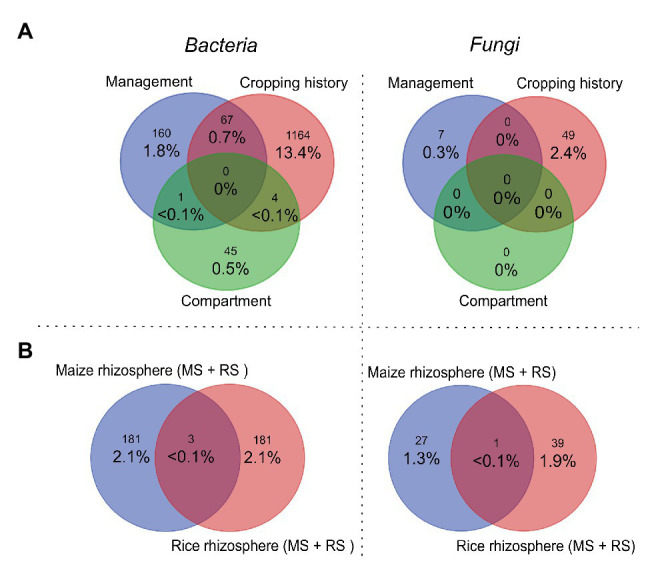
Venn diagrams displaying the percentages of bacterial (left) and fungal (right) OTUs and OTU numbers (small numbers) that were significantly affected in relative abundance in dependence on different grouping factors according to a STAMP analysis. **(A)** Displays the percentages and numbers of OTUs that were significantly affected by compartment, management (crop plant), and cropping history. Overlapping regions show numbers of those OTUs that were affected by two or even all three factors. Venn diagrams in **(B)** show percentages and numbers of OTUs that were significantly enriched in the rhizosphere vs. bulk soil comparatively for maize and rice. The rhizosphere-specific OTUs were identified for each plant independently in the two soils (MS and RS, considering only samples taken after 3 months) and then combined in one list per plant species to build the diagram. Percentages in all plots were calculated in relation to the total numbers of OTUs identified in this study (8,700 bacterial OTUs and 2,060 fungal OTUs). MS, maize soil; RS, rice soil.

We then evaluated how strongly the three main grouping factors (cropping history, management, and compartment) influenced the relative abundances of OTUs. Venn diagrams summarize numbers of significantly enriched OTUs in dependence on each of these grouping factors (Figure 3A). Cropping history affected 13.4% of all bacterial OTUs significantly, but only 2.4% of the fungal OTUs (Figure 3A). Management affected less OTUs (bacteria: 1.8%, fungi: 0.3%). The apparently weaker impact of management especially on the fungal OTUs appears to be in contrast to the responses seen in ADONIS and the NMDS plot. This is explained by a low number of responsive fungal OTUs that show strong shifts in relative abundance. Compartment related differences were linked to the lowest numbers of responsive OTUs with 0.5% of all bacterial OTUs, while no fungal OTUs were found to be compartment-specific (Figure 3A). We found some overlap between bacterial OTUs being affected by cropping history and flooding regime (0.7%), but no overlap between other factor combinations. No overlaps were observed for fungal OTUs (Figure 3A). The low numbers of OTUs responding in parallel to two different factors confirms the rather distinct impacts of cropping history and crop change on the microbial communities, as already suggested by the ADONIS autocorrelation results (Table 2).

### Compartment-Specific Microbial Changes in Beta Diversity in Response to Cropping History and Crop Change

To assess the role of cropping history and crop-rotational change specifically in the bulk soil and the rhizosphere, ADONIS was applied to the samples from each compartment independently. For the bacteria in the bulk soil cropping history remained clearly more influential (*R* ^2^ = 0.34, *p* = 0.001, Table 2) than changes in management regime (*R* ^2^ = 0.18, *p* = 0.001). The fungal community in the bulk soil was equally affected by cropping history (*R* ^2^ = 0.20, *p* = 0.001) and management (*R* ^2^ = 0.19, *p* = 0.001). A comparison of the effect of cropping history in dependence on management revealed that non-flooded soils were stronger separated due to cropping history (*R* ^2^ = 0.58, *p* = 0.001 for bacteria, *R* ^2^ = 0.38, *p* = 0.001 for fungi) than flooded soils (*R* ^2^ = 0.38, *p* = 0.001 for bacteria, *R* ^2^ = 0.26, *p* = 0.001 for fungi). To compare the effects of first-time flooding of MS with first-time upland cropping in RS, it was analyzed in MS and RS bulk soil independently. This revealed a comparable impact on the microbial communities in both soils upon the new management regime (*R* ^2^ = 0.37, *p* = 0.001 for bacteria and *R* ^2^ = 0.35, *p* = 0.001 for fungi in MS being first-time flooded vs. the non-flooded MS; *R* ^2^ = 0.38, *p* = 0.001 for bacteria in RS being non-flooded vs. the first-time flooded RS). This management response was only lower for fungi in RS upon the omission of flooding (*R* ^2^ = 0.23, *p* = 0.001). The overall effect of time remained low in the different data sub-sets (Table 2).

Analysis of all rhizosphere samples by ADONIS revealed an opposite trend as observed in bulk soil. The cultivated crop plant along with the management explained more of the variation in the data (*R* ^2^ = 0.37, *p* = 0.001 for bacteria; *R* ^2^ = 0.39, *p* = 0.001 for fungi) than cropping history, though cropping history remained relevant (with *R* ^2^ = 0.23, *p* = 0.001 for bacteria; *R* ^2^ = 0.19, *p* = 0.001 for fungi). The cropping history effect on rhizosphere samples was clearly visible in the NMDS plots (Figure 2, lower panels) with rice and maize rhizosphere samples being well separated. Separation due to the host plant in combination with the management regime was also clearly visible.

### Comparison of Taxa Responding to Cropping History and Altered Management Regimes in Bulk Soil

**Figure 4:**
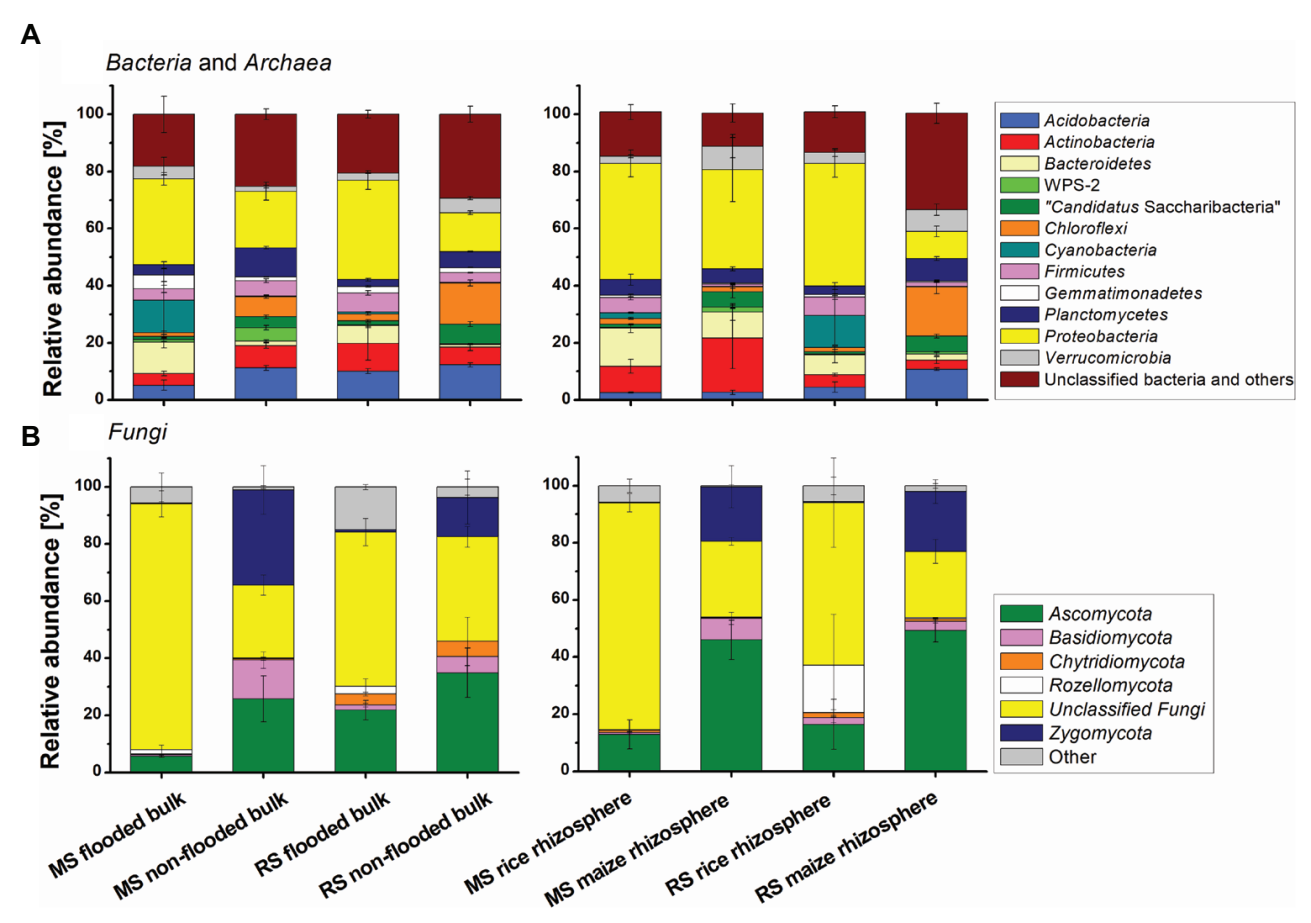
Differences in the **(A)** bacterial and archaeal and **(B)** fungal community composition in soils with different cropping history, management, and cultivated plant. Mean relative abundance values and SDs are shown for the dominant phyla. The group “other” includes all phyla with a relative abundance <2%. Bulk soil samples comprise all bulk soil samples taken after 3 months. MS, maize soil; RS, rice soil.

The composition of the microbial communities in the different soils was dominated by bacterial phyla that are commonly present in soils, including *Proteobacteria*, *Acidobacteria*, *Actinobacteria*, *Bacteroidetes*, *Planctomycetes*, *Firmicutes*, *Verrucomicrobia*, and *Chloroflexi*. Among the dominant fungal phyla were Ascomycota and Zygomycota (Figure 4). Effects of cropping history and altered cropping regimes on community composition were already detectable at phylum level. With regard to cropping history a significantly higher relative abundance of Proteobacteria, Bacteroidetes, and diverse unclassified fungi in flooded RS compared to the non-flooded MS was evident. In contrast, *Chloroflexi*, *Planctomycetes*, “*Candidatus* Saccharibacteria,” phyla WPS-1 and WPS-2 as well as *Basidiomycota*, *Glomeromycota*, and *Zygomycota* were significantly enriched in the non-flooded MS (Figure 4; Supplementary Table S1 for statistical analysis). Upon change in the management regime *Proteobacteria*, *Bacteroidetes*, *Gemmatimonadetes*, and further unclassified fungi became enriched in the flooded MS compared to the non-flooded MS. In the non-flooded RS, *Chloroflexi*, *Planctomycetes*, “*Candidatus* Saccharibacteria,” and phylum WPS-1 became enriched compared to the flooded RS. Thus, several of the phyla that were characteristic for a long-term management became enriched in the formerly differently managed soil upon change toward this management condition.

A more detailed analysis of the microbial community responses to cropping history and management was performed at OTU level. Specifically enriched and thus characteristic for a long-term managed RS (flooded) in comparison to MS (non-flooded) were 159 bacterial, but only four fungal OTUs (Supplementary Figure S1). Of these OTUs being characteristic for long-term continuous cropping in RS, none was increased in relative abundance when MS was flooded for the first-time compared to the non-flooded MS. In contrast, the majority of bacterial taxa (i.e., 46) being responsive to first-time flooding of MS showed a significant decrease in relative abundance. Typical OTUs being enriched in long-term managed RS vs. long-term MS were identified in a reduced dataset of abundant OTUs (Supplementary Table S3). Some *Bacteroidetes* (assigned to *Chitinophagaceae*, *Mucilaginibacter*, and *Pedobacter*) and different *Proteobacteria* (*Devosia*, *Rhodospirillaceae*, *Rhodocyclaceae*, and different unclassified *Betaproteobacteria*) were characteristic for RS, thus confirming results at phylum level. In addition, OTUs that were not seen to be responsive at phylum level were identified within the *Acidobacteria* (subgroup Gp1), *Actinobacteria* (*Gaiella*), *Chloroflexi* (*Anaerolineaceae*), *Firmicutes* (*Clostridiales*), and Chytridiomycota (*Spizellomyces*, *Chytridiomyces*) besides some unclassified fungal taxa (Supplementary Table S3). Of these, only very few members within the *Acidobacteria*, *Actinobacteria*, *Bacterioidetes*, and *Firmicutes* as well as an ascomycete (*Trichocomaceae*) were stimulated in MS upon first-time flooding (Supplementary Table S3). The majority of OTUs that responded to the management change in MS were not specifically enriched in the long-term managed RS.

Specifically enriched in long-term MS (non-flooded) in comparison to RS (flooded) were 124 bacterial and four fungal OTUs (Supplementary Figure S1). Of these, five bacterial OTUs were also enriched in RS when managed the under non-flooded conditions. Again, the majority of OTUs (i.e., 106) that responded to non-flooding of RS in comparison to regularly flooded RS were distinct from the taxa that characterize the long-term non-flooded MS. OTUs enriched in long-term non-flooded MS in comparison to flooded RS were *Actinobacteria* (*Gaiella*) some *Bacteroidetes* (*Cytophagales*), members of WPS-1, several OTUs of *Planctomycetes* (*Planctomycetales*), *Alphaproteobacteria* (especially *Rhizobiales*), unclassified *Betaproteobacteria*, rather unexpectedly some *Deltaproteobacteria* (*Desulfuromonadales*, *Mycococcales*, and *Syntrophobacter*), as well as different *Ascomycota*, *Basidiomycota* (*Tremellales*, *Cryptococcus*), and *Zygomycota* (especially *Mortierellales*; Supplementary Table S3). Of these, only the unclassified *Mortierellales* were at the same time strongly enriched in the non-flooded RS (Supplementary Table S3). The reason why this OTU was not seen as shared taxon in the Venn diagram (Supplementary Figure S1) is explained by the fact that data were analyzed by combining them in different ways.

### Identification of Host-Plant Specific OTUs in the Rhizosphere in Dependence on Cropping History

The composition of the microbial communities in the maize and rice rhizosphere was dominated by the same phyla that were predominant in bulk soil (Figure 4). Long-term continuous rice cropping stimulated the relative abundance of *Firmicutes* and unclassified fungi in the rhizosphere in comparison to continuous maize cropping. Both, groups were also significantly enriched upon first-time rice cultivation in MS (Supplementary Table S2). Long-term maize cropping increased the relative abundance of “*Candidatus* Saccharibacteria” phylum WPS-1, *Ascomycota*, *Basidiomycota*, and *Zygomycota* in comparison to continuous rice cropping (Figure 4). Among these phyla, “*Candidatus* Saccharibacteria,” Ascomycota, and Zygomycota were also enriched upon first-time maize cropping in RS (Supplementary Table S2).

To evaluate the specificity of the rhizosphere microbiota of rice vs. maize in detail, we identified the rhizosphere-enriched OTUs of both plants. Considering that the rhizosphere soil hosts taxa that remain present as part of a former bulk soil microbiota, we focused on those taxa that were significantly enriched in relative abundance in the rhizosphere soil of each plant species compared to the corresponding bulk soil. In the rice rhizosphere 181 bacterial OTUs were enriched, likewise as in the maize rhizosphere (i.e., 2.1% of the total number of bacterial OTUs detected in this study). Correspondingly, 27 fungal OTUs were detected in the rhizosphere of maize and 39 for rice. Only three bacterial and one fungal OTU were identified in the rhizosphere of both plant species (*Gemmatimonas*, *Cyanobacterium*, *Planctomycetaceae*, and *Penicillium*). Thus, the identity of rhizosphere specific OTUs of maize were mostly distinct from those of rice. Similarly, the identity of OTUs that were enriched in maize when grown in MS was mostly different to those being enriched in the rhizosphere when maize was grown in RS (15 bacterial and four fungal overlapping OTUs, Venn diagram not shown). This number was twice as high for bacteria in the rice rhizosphere, when the plant was grown in the two different soils, (31 bacterial but no fungal OTUs). These low numbers of shared OTUs in the rhizosphere of a plant when grown in two different soils (MS vs. RS) indicate that the assembly of the rhizosphere microbiota was strongly affected by the soil the plant was growing in.

**Figure 5:**
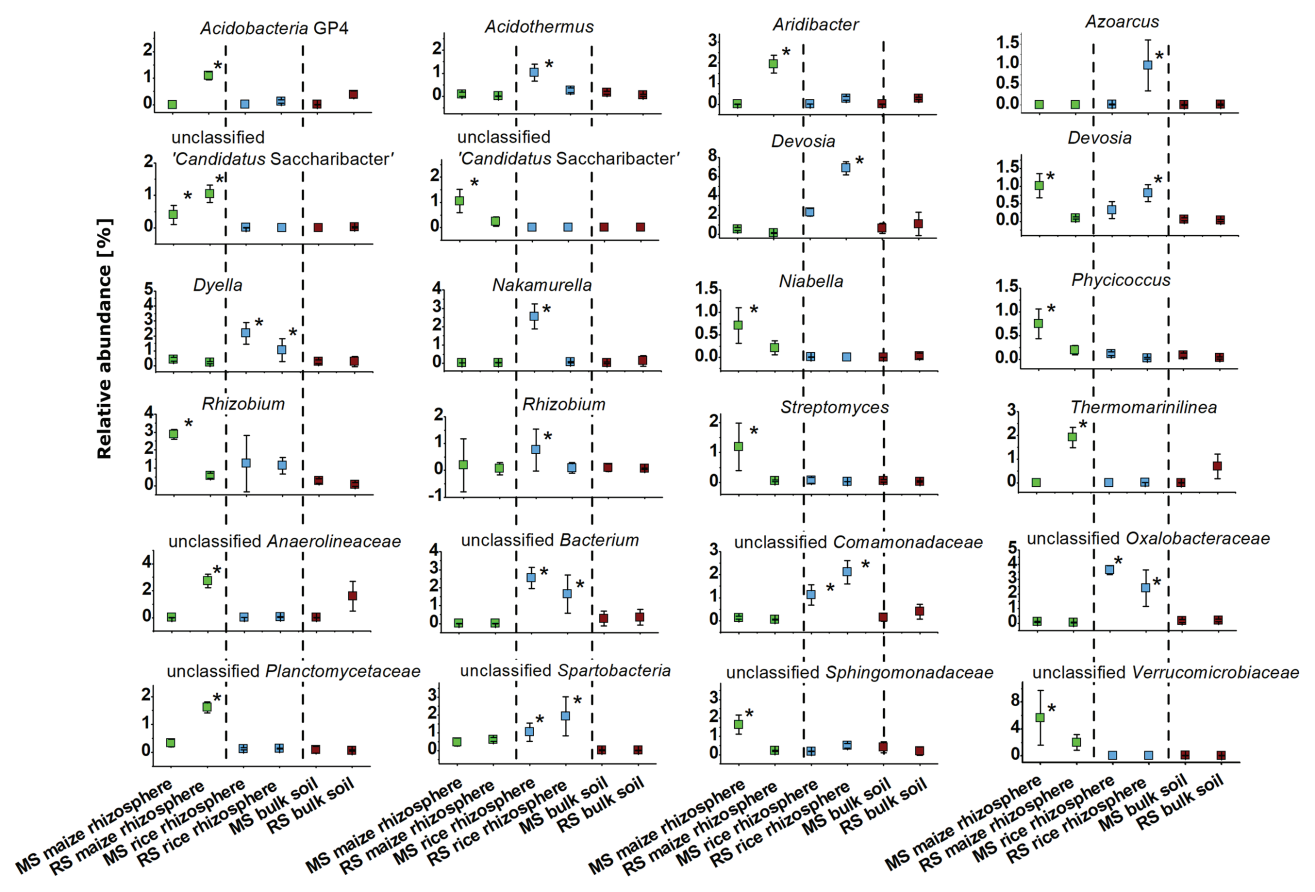
Dominant bacterial OTUs (>0.5% mean relative abundance among all rhizosphere samples) that were significantly enriched in the rhizosphere of maize or rice in comparison to bulk soil samples after 3 months. Rhizosphere samples of maize and rice are shown for plants grown in soil that underwent continuous maize cropping (MS, green) and rice cropping (RS, blue). Data for bulk soil samples were combined for MS and RS, respectively, and are displayed for comparison (brown). Significant differences with *p* < 0.05 between these six groups of samples according to ANOVA with *post hoc* tests are indicated by *.

**Figure 6:**
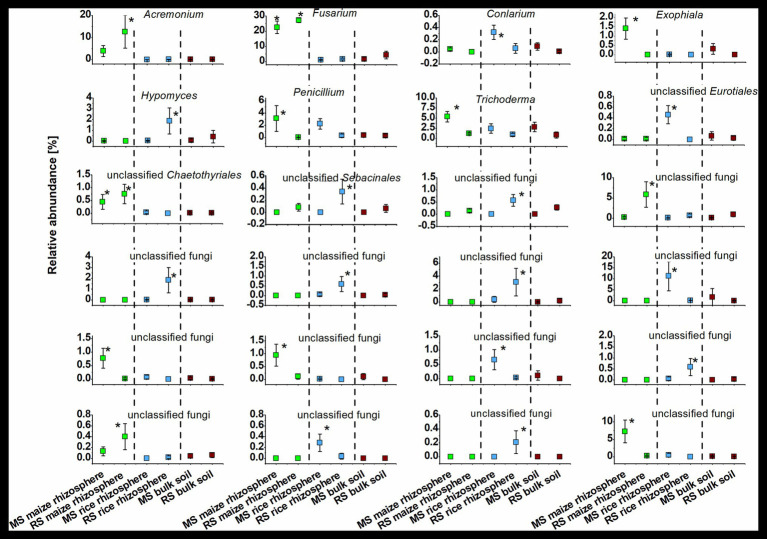
Dominant fungal OTUs (>0.3% mean relative OTU abundance among all rhizosphere samples) that were significantly enriched in the rhizosphere of maize or rice in comparison to bulk soil samples after 3 months. Rhizosphere samples of maize and rice are shown for plants grown in soil that underwent continuous maize cropping (MS, green) and rice cropping (RS, blue). Data for bulk soil samples were combined for MS and RS, respectively, and are displayed for comparison (brown). Significant differences with *p* < 0.05 between these six groups of samples according to ANOVA with *post hoc* tests are indicated by *.

The identity of taxa that were specifically enriched in the rhizosphere of a plant species was evaluated in detail by direct comparisons of relative abundances between samples, thereby focusing on the most abundant OTUs (Figures 5, 6). Only a few occurred in the rhizosphere of a plant species in both soils, underlining the distinctiveness of the rhizosphere microbiota in dependence on the soil. In rice these common OTUs were members of *Dyella* and an unclassified OTU of *Oxalobacteraceae*, *Comamonadaceae*, *Spartobacteria*, and *Bacteria*. In maize these were besides an unclassified member of “*Candidatus* Saccharibacteria” fungi of the genus *Fusarium* and an unclassified *Chaetothyriales*. To evaluate take-over effects in the rhizosphere upon crop change, OTUs were identified that were enriched in the maize rhizosphere in RS but not in MS. These were *Acidobacteria* group GP4, *Aridibacter*, *Thermomarinilinea*, unclassified *Anaerolineaceae*, unclassified *Planctomycetes*, as well as *Acremonium* and two OTUs assigned to unclassified fungi. Vice versa, a few OTUs were found enriched in the rice rhizosphere in MS compared to the rice rhizosphere in RS (*Acidothermus*, *Nakamurella*, *Rhizobium*, as well as *Conlarium*, unclassified *Eurotiales*, and three further unclassified fungal OTUs). None of these OTUs that were enriched in the rhizosphere when the host plant was grown for the first-time in a soil of different cropping history was characteristic for the rhizosphere of the other host plant or of high abundance in the respective bulk soil. This shows that rhizosphere-specific OTUs were not taken over from the rhizosphere specific microbiota of the other crop plant.

## Discussion

### Effect of Continuous Cropping and First-Time Crop Change on Bacterial and Fungal Alpha Diversity in Bulk Soil

Long-term continuous rice and maize cropping resulted in a higher bacterial and fungal richness and higher bacterial evenness in RS than in MS (Table 1). This indicates that regular flooding and rice cultivation over years increased the microbial diversity, as already seen in earlier studies (Yang and Zhang, 2014; Hou et al., 2018; Maarastawi et al., 2018). Regular flooding and drainage may create more different habitats in rice fields that may host more diverse microbial communities. This is possibly linked to a greater microbial seed bank in the soil with microbes being partly well-adapted to oxic and partly to anoxic conditions. This increase in diversity also applies to the fungal community, although it is often assumed that the majority of fungi cannot survive in water-logged or anoxic environments (Wurzbacher et al., 2010; Grahl et al., 2012), which would suggest a lower diversity in paddy soils. However, despite the increased diversity we observed, the fungal population size may actually be reduced in paddy soils, which should be evaluated in future studies. Along with physiological studies, this could show whether fungal populations are better adapted to regular anoxic conditions in soils than often thought.

First-time crop change caused responses in the bulk soil microbiota by the new management regimes, primarily related to the altered flooding conditions. We expected to see an increase in microbial diversity in MS upon first-time flooding and a decrease in RS upon extended non-flooding. Instead, we observed mostly declines in the alpha diversity measures, i.e., a decrease of bacterial and fungal evenness in flooded MS and a decline of bacterial and fungal richness and fungal evenness in non-flooded RS (Figure 1). The diversity decline in paddy soil upon upland cropping may be seen as a first step toward the development of an upland soil microbial community. That the changes remained rather slight is likely due to the fact that paddy soils undergo regular drainage anyway, resulting in a low reactivity in case the non-flooded period is extended over one cropping season. The observed decline of some diversity measures in MS upon first-time flooding was in contrast to our expectation. The introduction of flooding probably represents an ecosystem disturbance with declining diversity in a first instance, especially for fungi, rather than a straight development toward a more complex paddy soil community. In other studies, different results were seen, reporting a lower or higher microbial diversity after flooding of an upland soil (Li et al., 2020; Zhu et al., 2020). We thus conclude that the higher diversity that is often observed in paddy soils compared to upland soils develops after a possible initial decline in diversity, which is considered to be a response to the disturbance by the altered cropping conditions, especially the flooding.

The microcosm experiment under flooded conditions was performed 2 years later than the non-flooded experiment. The longer soil storage period may have affected our findings due to a decline in alpha diversity over time. If this had been a major problem, we should have seen a reduced diversity in the flooded RS and MS compared to the corresponding non-flooded soils, but this was not the case (Figure 1). In case of minor storage effects, the observed declines in alpha diversity upon extended non-flooding of RS would have been stronger than observed, while the decline in fungal diversity upon first-time flooding of MS would actually have been weaker. Despite this remaining uncertainty regarding the strength of the response, we are confident that our findings along with the literature knowledge lead to a valid conclusion.

### Responses of the Microbial Communities in Bulk Soils to Cropping History and Flooding

Continuous rice and maize cultivation resulted in clear differences in the microbial community composition in the respective soils (Figure 2; Table 2 bulk soil dataset) with ~14% of all bacterial OTUs and 2.4% of all fungal OTUs responding to cropping history (Figure 3A). Physico-chemical differences that established in these soils over years are likely responsible for this effect. A higher availability of iron, phosphate, and manganese and a higher pH was found in RS in comparison to MS in a study performed a few years earlier at the same study site (Houtermans and Lehndorff, personal communication). The regular flooding of paddy soils can be considered as major cause for the differences. It leads to strong changes in oxygen availability in the soil, therewith causing changes in soil chemical properties (Liesack et al., 2000; Schmidt et al., 2011 and references within) and inducing rapid and drastic microbial community changes, as e.g., observed by Zhu et al., (2020). In addition, further crop-specific management practices as well as the cultivated crop plant itself introduce changes in the soil microbial community, as discussed previously (Maarastawi et al., 2018).

The altered management regime upon a change in crop cultivation affected the bacterial communities in MS and RS almost at equal strength, while the fungal communities were affected more strongly upon flooding of MS than in RS upon non-flooding (Table 2, bulk soil datasets of MS and RS). Such a fungal response can be expected as most fungi are aerobic or micro-aerophilic (e.g., Wurzbacher et al., 2010; Grahl et al., 2012). We observed a particular strong decrease of two OTUs representing members of the *Mortierellales* upon flooding of MS (from 20.5 to 0.5% and from 11.1 to 0.1% relative abundance, Supplementary Table S3). Members of the *Mortierellales* are known to occur at high relative abundance in some upland soils and are involved in the degradation of plant residue (España et al., 2011; Zhang et al., 2020). Moreover, *Mortierella* species have been reported to be involved in plant-residue derived carbon degradation under non-flooded conditions in paddy soil (Murase et al., 2012) and are considered to be fast-growing decomposers (España et al., 2011). This ecological profile corresponds well to their presence in MS and the rapid appearance of one of these OTUs upon first-time upland cropping in RS. Also remarkable, first-time flooding of MS increased the relative abundance of diverse unclassified fungi (Figure 4). This might be the result of a dieback of several classified taxa. The unclassified fungal groups may be of specific relevance in paddy soils, because a high relative abundance was also seen in the long-term flooded RS, while they were of minor relevance in the non-flooded MS and the maize rhizosphere. The biology of these unclassified fungi remains currently unknown and therewith their possible roles and adaptations to anoxic conditions. This deserves more attention in future studies.

The soil microbiota reached an intermediate compositional state after the management change, as evident from the distinct clustering of the samples undergoing a new crop management regime compared to those with long-term continuous cropping management (Figure 2). This is in line with our expectation that the bulk soil microbiota responds to an altered flooding regime by a partial transition of the community composition during the first season. We expected a direct development toward the community seen in the corresponding long-term managed soil. Instead, we observed a different development, with OTUs increasing in relative abundance that were largely distinct from those being characteristic for both continuous cropping regimes. The introduction of a flooding regime as well as its omission does apparently not directly lead to a microbial community that is characteristic for a soil that undergoes this management regime in the long-term. Moreover, these changes did not become evident at phylum level, where the results suggested a direct development of the microbial communities to the opposite type of microbiota, but became visible at higher taxonomic resolution, indicating that specific taxa within the phyla responded differently, as e.g., seen for the *Bacteroidetes* or *Proteobacteria* (compare Figure 4 with data in Supplementary Table S3).

The bacterial and fungal communities in the two flooded bulk soils were, despite their strong separation, more similar to each other than communities in the two non-flooded soils in the NMDS plots (Figure 2 and in Table 2, datasets of flooded and non-flooded bulk soils), i.e., the impact of cropping history remained stronger in non-flooded than in flooded soils. These findings suggest that the pace of transition from one type of bulk soil to another is dependent on the management, i.e., flooded soils appear to align more rapidly than non-flooded soils. Regarding this point, it again has to be considered that the microcosm experiments were conducted with a time delay. The longer storage period of soils that were flooded may have caused a slowdown of microbial reactivity. However, since we observed stronger changes in the flooded soils compared to the non-flooded soils, the response to flooding was indeed stronger than to the omission of flooding.

### Responses of the Rhizosphere Microbiota to Cropping History and Crop Rotation

Bacterial and fungal richness, though not evenness, were strongly affected by cropping history in the rhizosphere. This influence has already been discussed by Maarastawi et al. (2018) for the introduction of maize in RS. The complementary set-up with rice cultivation in MS revealed now contrasting responses. First-season rice growth in MS caused a decrease in bacterial and fungal richness in the rice rhizosphere compared to growth in RS, while the richness of bacteria and fungi of the maize rhizosphere increased when grown in RS vs. MS (Figure 1). Evenness remained largely unaffected. Based on these findings, we cannot fully validate our hypothesis stating that diversity increases in the rhizosphere of a crop plant if cultivated in a soil that has not yet been used to cultivate this crop species before. We assumed this to occur due to a more unspecific selection process of microorganisms in the rhizosphere, resulting from a lower number of well-adapted microbial strains in a soil in which the plant has not been grown before. This may apply to some extent in the maize rhizosphere, but not in the rice rhizosphere, where diversity declined upon growth in MS. The rhizosphere microbiota is apparently dominantly affected by the type of soil the plant is growing in, which is primarily defined by cropping history in this study.

Regarding community composition, the rhizosphere microbiota differed more strongly between the two cultivated crops than in dependence on the cropping history of the soils in which the plants were grown (Table 2; Figure 2). These differences are likely resulting from plant-specific rhizodeposition, leading to the enrichment of specific microorganisms in the rhizosphere. Moreover, the crop-specific management regimes, especially the flooding, cause changes in redox conditions and thus alter chemical processes in the rhizosphere (Gao et al., 2002; Bais et al., 2006; Schmidt et al., 2011; Arruda et al., 2013; Berg et al., 2017; Maarastawi et al., 2018). To some extent, the observed differences in the rhizosphere microbiota related to crop plant species might also be the result of differences in the experimental setup and sample collection protocols for rhizosphere samples. We cannot fully exclude a bias resulting from these differences, but it is very unlikely that the strong differences observed between the maize and rice rhizosphere microbiota are primarily resulting from these methodological differences.

In the NMDS plot, bacterial communities of the rice rhizosphere clustered more closely together than those of the maize rhizosphere, indicating that they converged faster in the rice rhizosphere than in the maize rhizosphere (Figure 2). This pattern was also observed in the bulk soil samples as discussed before, so it may be related to flooding. A possible explanation for this pattern may lay in the change of physicochemical conditions at the small scale (reviewed in Tecon and Or, 2017). Non-flooded soils are known to be spatially patchy, and with decreasing water availability mobility and dispersal of microbes becomes limited and micro-habitats more separated. The flooding of soils reconnects these micro-habitats, allowing better mobility and therefore possibly a more rapid migration. This may support a faster adjustment of the microbial community to environmental conditions and explain the more similar microbial community patterns in the rice rhizosphere as well as in bulk soil in the flooded soils.

### Influence of Previously Cultured Crops on the Recruitment of Specific Microorganisms in the Rhizosphere

The observed impact of cropping history and management regime demonstrates that these provide an important environmental framework for microbial communities in the rhizosphere, but the plant species itself is largely responsible for the selection of a specific rhizosphere microbiota. In this study, the rhizospheres of rice and maize shared only very few species (Figure 3B), which points to a high selectivity regarding the recruitment of microorganisms. Surprisingly and in contrast to our hypothesis, we did not find many shared taxa and thus a take-over effect of the rhizosphere microbiota when maize was first-time cultivated in RS or rice was introduced in MS. Apparently, the differences in the rhizospheres of these two plants are substantial, leading to the enrichment of different microorganisms. This may be caused by differences in the composition of root exudates between C_3_ (rice) and C_4_ (maize) plants, with higher amounts of amino acids and organic acids in C_3_ plants compared to higher concentrations of carbohydrates in C_4_ plants (reviewed in Vranova et al., 2013). Furthermore, the oxygen availability in the rice rhizosphere is spatially and temporarily highly variable and oxygen depletion likely creates different habitats (Schmidt et al., 2011). The strong differences are also related to the fact that we focused on microbial taxa that were specifically enriched in the rhizosphere of a plant in comparison to the corresponding bulk soil in which the plant was grown. Thus, we excluded taxa from our comparison that occur in the rhizosphere of both plants without being significantly enriched by the plant, as we considered those as not highly rhizosphere-specific.

We also observed remarkable differences between the rhizosphere communities of a crop species in dependence on the cropping history of a soil. The introduction of a new plant species in a continuous cropping system apparently leads to the recruitment of several previously rare taxa in the rhizosphere of the new plant (Figures 5, 6). OTUs assigned to Acidobacteria subgroup GP4, *Aridibacter*, *Thermomarinilinea*, *Anaerolineaceae*, *Planctomycetaceae*, *Acremonium*, and an unclassified fungal OTU were highly enriched in the rhizosphere of maize grown in RS but not in MS or any other group of samples. Likewise, *Acidothermus*, *Nakamurella*, *Rhizobium*, *Conlarium*, an unclassified *Eurotiales*, and several other unclassified fungi were specifically enriched in the rice rhizosphere when grown in MS. A crop species that is newly introduced into a soil obviously recruits other microorganisms compared to a soil in which it has been cultivated for decades. The development of a highly characteristic host-plant microbiome may need a succession over more than one growth period. Such a succession is possibly also dependent on specific physico-chemical properties in the rhizosphere soil, which will also need to undergo a succession process. This can at least be considered for this study, because the cultivation conditions for maize and rice differ substantially. A pronounced enrichment of potential plant beneficial microorganisms under the new soil conditions was not evident. Among the enriched taxa we identified, such traits are primarily known for *Rhizobium* (Garrido-Oter et al., 2018). The plants do apparently not acquire more beneficials when growing in a soil not yet adapted to host this plant species. These microorganisms may also have to be enriched over longer periods of time.

## Conclusion

We disentangled the effects of cropping history, management regime, which is primarily defined by flooding, and cultivated plant species in rice and maize cropping systems. We therefore performed microcosm experiments with soils from neighboring fields under long-term continuous rice and maize cultivation. Cropping history was identified as a main factor shaping microbial community composition in bulk and rhizosphere soil under these contrasting conditions. This impact remained still clearly visible after one season of crop change, even though the introduction of a new crop species into the rice and maize field soils caused major changes in the rhizosphere and bulk soil microbiota. However, the communities did not develop straight toward those known from long-term continuous cropping conditions. One season of crop change is not sufficient for the microbiota to convert toward the characteristic microbiota seen under long-term continuous cropping, which we considered as blueprint of a well-adapted microbiome for rice and maize cropping here. Instead, the introduction of a new plant species in a continuous cropping system promoted some previously rare taxa. Thus, the succession of the rhizosphere and bulk soil microbiota from one system to another occurs *via* a distinct intermediate stage. The rhizosphere microbiome is shaped by the host plant-specific factors and crop management regime. Although the cultivation history plays a role, the previously cultivated crop species does not strongly influence the rhizosphere microbiome of the new crop. This is obvious from the non-existing overlap of shared taxa between the two plant species. Plants do apparently not strongly benefit from the microbiota of the previous crop, at least not in case of rice and maize, which are characterized by quite distinct conditions in their rhizospheres.

## Data Availability Statement

The datasets presented in this study can be found in online repositories. The names of the repository/repositories and accession number(s) can be found at: https://www.ebi.ac.uk/ena, PRJEB23682; https://www.ebi.ac.uk/ena, PRJEB35017.

## Author Contributions

KF and CK conceived and designed the research and wrote the paper. KF and SZ performed the experiments. KF and CK analyzed the data. All authors contributed to the article and approved the submitted version.

### Conflict of Interest

The authors declare that the research was conducted in the absence of any commercial or financial relationships that could be construed as a potential conflict of interest.
