# Supplementary material for: Development of a Distinct Microbial Community Upon First Season Crop Change in Soils of Long-Term Managed Maize and Rice Fields
Source: Front Microbiol. 2020 Nov 9;11:588198. doi: 10.3389/fmicb.2020.588198 (PMC7680734; doi:10.3389/fmicb.2020.588198)
Supplement: Supplementary file 1 [file Data_Sheet_1.pdf]

Supplementary Material

Development of a distinct microbial community upon first season crop change in soils of long-term managed maize and rice fields

Katharina Frindte*, Sarah A. Zoche & Claudia Knief

Correspondence: kfrindte@uni-bonn.de

1 Supplementary Figures and Tables

1.1 Supplementary Figures

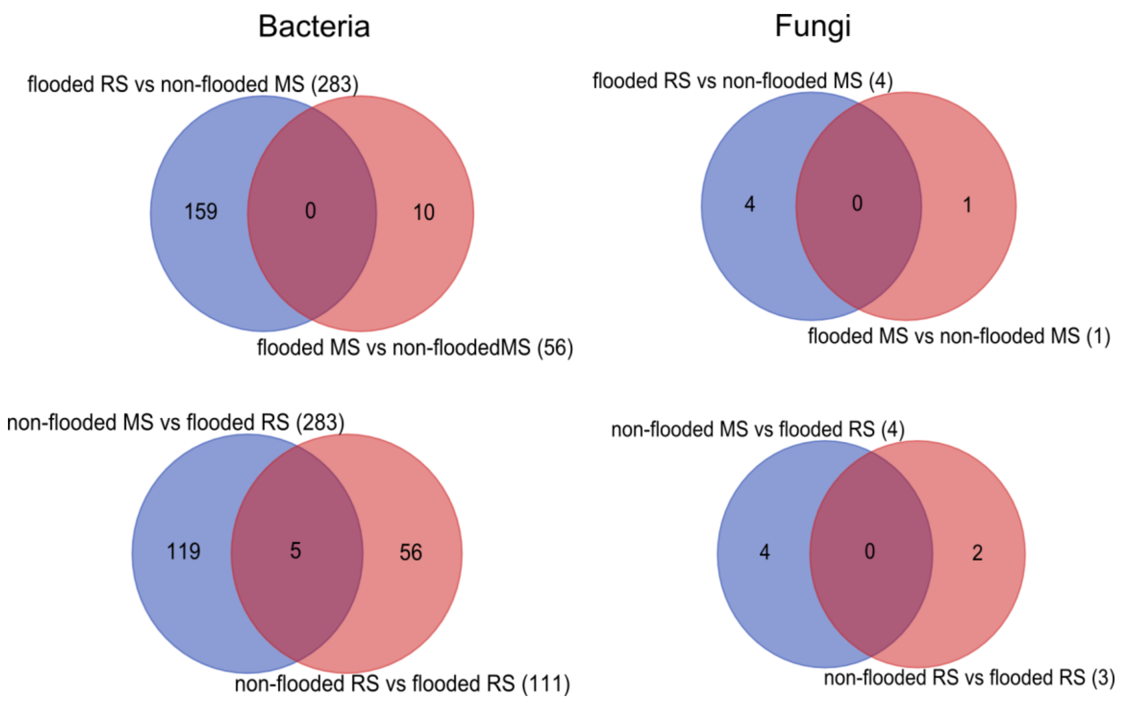

Figure S1. Venn diagrams showing the overlap between a microbiota being adapted to long-term continuous cropping and the OTUs that occur after first time crop cultivation in a formerly differently managed soil. Diagrams in A.) show number of bacterial (left) and fungal (right) OTUs that were typical in flooded RS, i.e. significantly enriched in flooded RS in comparison to the non-flooded MS (in blue). In red, OTUs are shown that were significantly enriched in first-time flooded MS in comparison to the non-flooded MS. The overlap represents OTUs that enrich upon first-time flooding in MS and are characteristic for long-time flooded RS soils. Diagrams in B.) show the number of bacterial (left) and fungal (right) OTUs that were typical for non-flooded MS, i.e. significantly enriched in non-flooded MS in comparison to the flooded RS (in blue). In red, OTUs are shown that were significantly enriched in first-time non-flooded RS in comparison to flooded RS. The overlap represents OTUs that enrich upon first-time non-flooding of RS and that are characteristic for long-term non-flooded MS. The total number of OTUs being responsive to a change in long-term cropping regime (blue) or short-term flooding regime (red) is given in brackets behind each comparison. MS = maize soil, RS= rice soil.

1.2 Supplementary Tables

Table S1: Bacterial and fungal phyla showing significant differences in relative abundance (%) in the bulk soil samples in dependence on the experimental treatments. Only phyla with a mean relative abundance > 1 % in at least one treatment were included in the table. Responsive phyla were identified based on ANOVA with Tukey-Kramer Post-hoc tests and Benjamini-Hochberg FDR correction. MS = maize soil, RS = rice soil.

Domain	Phylum	MS flooded	MS non-flooded	RS flooded	RS non-flooded
Bacteria	*Acidobacteria*	5.2 ± 1.8[b]	11.2 ± 0.91[a]	10.1 ± 0.9[a]	12.3 ± 0.7[a]
	Bacteriodetes	11.0 ± 2.0[a]	1.6 ± 0.4[c]	6.2 ± 0.8[b]	1.0 ± 0.2[c]
	'*Candidatus* Saccharibacteria'	1.1 ± 0.6[c]	3.8 ± 0.6[b]	1.5 ± 0.5[c]	6.8 ± 0.9[a]
	Chloroflexi	1.3 ± 0.5[c]	7.0 ± 0.6[b]	2.4 ± 0.4[c]	14.4 ± 1.1[a]
	Firmicutes	3.9 ± 1.3[b]	5.3 ± 0.7[ab]	6.7 ± 0.8[a]	3.3 ± 0.1[b]
	Gemmatimonadetes	4.8 ± 2.2[a]	1.3 ± 0.2[b]	2.4 ± 0.3[ab]	1.8 ± 0.1[b]
	Planctomycetes	3.6 ± 1.2[c]	10.2 ± 0.5[a]	2.3 ± 0.5[c]	5.6 ± 0.2[b]
	Proteobacteria	30.1 ± 2.3[a]	19.8 ± 3.1[b]	34.9 ± 3.3[a]	13.6 ± 0.6[b]
	WPS-1	0.99 ± 0.23[bc]	1.9 ± 0.15[a]	0.7 ± 0.1[c]	1.2 ± 0.1[b]
	WPS-2	0.99 ± 0.7[b]	4.7 ± 0.8[a]	0.2 ± 0.1[b]	0.3 ± 0.1[b]
Eukarya	*Ascomycota*	5.7 ± 0.5[b]	25.7 ± 8.0[a]	21.9 ± 3.4[ab]	34.9 ± 8.6[a]
	Basidiomycota	0.5 ± 0.1[b]	13.6 ± 2.9[a]	1.8 ± 0.6[b]	5.8 ± 3.1[b]
	Glomeromycota	0.0 ± 0.0[b]	0.3 ± 0.2[a]	0.0 ± 0.0[b]	0 ± 0.0[b]
	unclassified fungi	86.2 ± 4.6[a]	25.6± 3.6[d]	54.0 ± 4.8[b]	36.6 ± 3.6[c]
	Zygomycota	0.2 ± 0.1[b]	33.4 ± 8.5[a]	0.8 ± 0.1[b]	13.7 ± 9.4[b]

Table S2: Bacterial and fungal phyla showing significant differences in relative abundance (%) in the rhizosphere samples in dependence on the experimental treatments. Only phyla with a mean relative abundance > 1 % in at least one treatment were included in the table. Responsive phyla were identified based on ANOVA with Tukey-Kramer Post-hoc tests and Benjamini-Hochberg FDR correction. Superscript letters indicate significant differences between the samples. MS = maize soil, RS = rice soil.

**Domain**	**Phylum**	**MS rice rhizosphere**	**MS maize rhizosphere**	**RS rice rhizosphere**	**RS maize rhizosphere**
Bacteria	*Acidobacteria*	2.6 ± 0.2^{b}	2.6 ± 0.7^{b}	4.5 ± 1.8^{b}	10.8 ± 0.5^{a}
	Armantimonadetes	1.0 ± 0.3^{a}	0.1 ± 0.1^{c}	0.3 ± 0.1^{c}	0.8 ± 0.1^{ab}
	Bacteriodetes	13.3 ± 1.5^{a}	9.1 ± 2.9^{a}	7.1 ± 2.8^{ab}	2.1 ± 0.6^{b}
	'Candidatus Saccharibacteria'	1.0 ± 0.2^{b}	5.3 ± 2.2^{a}	0.8 ± 0.1^{b}	5.5 ± 0.7^{a}
	Chloroflexi	2.1 ± 0.3^{b}	1.8 ± 0.5^{b}	1.5 ± 0.6^{b}	17.2 ± 2.4^{a}
	Firmicutes	5.3 ± 0.8^{a}	0.9 ± 0.3^{b}	6.5 ± 0.9^{a}	1.4 ± 0.1^{b}
	Planctomycetes	5.5 ± 2.0^{ab}	4.9 ± 0.7^{ab}	3.0 ± 1.2^{b}	7.9 ± 0.7^{a}
	Proteobacteria	40.6 ± 4.7^{a}	34.7 ± 11.3^{a}	42.8 ± 4.8^{a}	9.5 ± 1.9^{b}
	WPS-1	0.3 ± 0.1^{b}	0.7 ± 0.2^{b}	0.2 ± 0.1^{b}	1.3 ± 0.1^{a}
	WPS-2	0.3 ± 0.0^{b}	1.8 ± 0.6^{a}	0.2 ± 0.1^{b}	0.8 ± 0.2^{b}
Eukarya	*Ascomycota*	12.9 ± 5.1^{b}	46.0 ± 6.9^{a}	16.4 ± 8.7^{b}	49.3 ± 4.0^{a}
	Basidiomycota	0.6 ± 0.3^{b}	7.6 ± 2.1^{a}	2.4 ± 1.8^{b}	3.2 ± 0.5^{b}
	unclassified fungi	79.5 ± 3.2^{a}	26.7 ± 1.3^{b}	57.0 ± 15.6^{a}	23.3 ± 4.2^{b}
	Zygomycota	0.3 ± 0.1^{b}	19.1 ± 7.4^{a}	0.5 ± 0.1^{b}	21.0 ± 4.2^{a}

Table S3: Dominant fungal and bacterial OTUs (> 0.5 % for bacteria and 0.3 % for fungi) that were significantly enriched in the bulk soil in comparison to rhizosphere samples after 3 months in dependence on the cropping history and flooding. Responsive phyla were identified based on ANOVA with Tukey-Kramer Post-hoc tests and Benjamini-Hochberg FDR correction. Superscript letters indicate significant differences between samples. Data for rhizosphere samples (not shown) were combined for rice and maize, respectively, and included in ANOVA with the aim to identify and exclude taxa that were more characteristic for rhizosphere soil. Highlighted in green are taxa that were significantly enriched in RS flooded as well as MS flooded compared to MS non-flooded. Highlighted in orange are taxa that were significantly enriched in MS non-flooded and RS non-flooded compared RS flooded. These represent OTUs that point to a direct development of the microbiota towards the community seen in long-term managed soils. MS = maize soil, RS = rice soil, uncl. = unclassified.

**Domain**	**Phylum**	**Last identified phylogenetic level**	**MS flooded**	**MS non-flooded**	**RS flooded**	**RS non-flooded**
Bacteria	*Acidobacteria*	uncl. Gp1	0 ± 0^{c}	0 ± 0^{c}	0.2 ± 0^{b}	0.6 ± 0.1^{a}
		uncl. Gp1	0 ± 0^{b}	0 ± 0^{b}	0 ± 0^{b}	0.8 ± 0.1^{a}
		uncl. Gp1	0.7 ± 0.4^{ab}	0.1 ± 0^{c}	1.2 ± 0.2^{a}	0.3 ± 0.1^{b}
		uncl. Gp1	1.2 ± 0.2^{ab}	1.6 ± 0.3^{a}	1.2 ± 0.2^{ab}	0.6 ± 0.1^{b}
		uncl. Gp1	0.9 ± 0.1^{a}	0 ± 0^{b}	0 ± 0^{b}	0 ± 0^{b}
	Actinobacteria	*Gaiella occulta*	0.2 ± 0.1^{b}	0.6 ± 0.1^{a}	0 ± 0^{b}	0 ± 0^{b}
		Gaiella occulta	0.7 ± 0.3^{a}	0.1 ± 0^{b}	0.5 ± 0.2^{ab}	0.1 ± 0^{b}
		Gaiella occulta	2.7 ± 1.5^{a}	0 ± 0^{b}	0.1 ± 0^{b}	0 ± 0^{b}
	Bacteriodetes	uncl. *Cytophagales*	0.1 ± 0.1^{b}	0.5 ± 0.1^{a}	0 ± 0^{b}	0 ± 0^{b}
		uncl. *Cytophagales*	0.1 ± 0.1^{b}	0.7 ± 0.2^{a}	0 ± 0^{b}	0.3 ± 0.1^{b}
		uncl. *Cytophagales*	0.1 ± 0^{b}	1 ± 0.1^{a}	0.1 ± 0^{b}	0.2 ± 0^{b}
		uncl. *Chitinophagaceae*	1 ± 0.6^{a}	0 ± 0^{b}	0.9 ± 0.1^{a}	0.1 ± 0^{b}
		uncl. *Mucilaginibacter*	0.3 ± 0.2^{bc}	0 ± 0^{c}	0.8 ± 0.1^{a}	0.4 ± 0.1^{b}
		Pedobacter ginsenosidimutans	0.4 ± 0.4^{ab}	0 ± 0^{b}	0.5 ± 0.3^{a}	0 ± 0^{b}
		uncl. *Pedobacter*	1.2 ± 0.7^{a}	0.3 ± 0.1^{b}	0 ± 0^{b}	0 ± 0^{b}
	Candidate division WPS-1	uncl.	0.1 ± 0.1^{b}	0.7 ± 0.1^{a}	0 ± 0^{b}	0 ± 0^{b}
	Candidatus Saccharibacteria	uncl.	0 ± 0^{b}	0 ± 0^{b}	0 ± 0^{b}	0.8 ± 0.3^{a}
	Chloroflexi	uncl. *Anaerolineaceae*	0 ± 0^{b}	0 ± 0^{b}	0.1 ± 0^{b}	0.6 ± 0.1^{a}
		uncl. *Anaerolineaceae*	0 ± 0^{b}	0 ± 0^{b}	0.1 ± 0^{b}	1.3 ± 0.2^{a}
		uncl. *Anaerolineaceae*	0 ± 0^{c}	0 ± 0^{c}	0.3 ± 0.1^{b}	2.6 ± 0.1^{a}
		uncl. *Anaerolineaceae*	0 ± 0^{b}	0 ± 0^{b}	0 ± 0^{b}	0.8 ± 0.1^{a}
	Cyanobacteria	uncl. *Cyanobacteria*	0 ± 0^{b}	0 ± 0^{b}	0.1 ± 0^{b}	1 ± 0.1^{a}
		uncl. *Cyanobacteria*	0 ± 0^{c}	0.1 ± 0^{bc}	0.1 ± 0^{b}	0.9 ± 0.1^{a}
		uncl. *Cyanobacteria*	0 ± 0^{c}	0 ± 0^{c}	0.4 ± 0.1^{b}	1.2 ± 0.2^{a}
	Deinococcus	uncl. *Deinococcus*	0.3 ± 0.1^{b}	0.8 ± 0.2^{a}	0.3 ± 0.1^{b}	0.4 ± 0.1^{b}

Domain	Phylum	Last identified phylogenetic level	MS flooded	MS non-flooded	RS flooded	RS non-flooded
Bacteria	*Firmicutes*	*Bacillus kokeshiiformis*	1 ± 0.3[b]	1.5 ± 0.2[a]	1 ± 0.2[ab]	0.3 ± 0.1[c]
		uncl. *Paenibacillus*	0.7 ± 0.3[a]	0.1 ± 0[b]	0.2 ± 0[b]	0 ± 0[b]
		uncl. *Ruminococcaceae*	0.5 ± 0.5[a]	0 ± 0[b]	0 ± 0[b]	0 ± 0[b]
		uncl. *Clostridiales*	0.6 ± 0.2[a]	0.1 ± 0[b]	0.6 ± 0.1[a]	0.2 ± 0.1[b]
		uncl. *Clostridiales*	0.5 ± 0.2[a]	0 ± 0[b]	0.2 ± 0[b]	0 ± 0[b]
		uncl. *Clostridiales*	0 ± 0[b]	0 ± 0[b]	0.1 ± 0[b]	0.9 ± 0.2[a]
		uncl. *Firmicutes*	0 ± 0[b]	0 ± 0[b]	0.2 ± 0[b]	1.3 ± 0.1[a]
	Gemmatimonadetes	*Gemmatimonas aurantiaca*	0 ± 0[b]	1.2 ± 0.2[a]	0 ± 0[b]	0.2 ± 0[b]
		Gemmatimonas aurantiaca	0.1 ± 0[b]	0.5 ± 0.1[a]	0 ± 0[b]	0.1 ± 0.1[b]
	Planctomycetes	uncl. *Planctomycetaceae*	0 ± 0[c]	0.5 ± 0.1[a]	0 ± 0[c]	0.4 ± 0.1[b]
		uncl. *Planctomycetaceae*	0 ± 0[b]	0.6 ± 0.1[a]	0 ± 0[b]	0.1 ± 0[b]
		uncl. *Planctomycetaceae*	0.1 ± 0.1[b]	1.5 ± 0.3[a]	0 ± 0[b]	0 ± 0[b]
		uncl. *Planctomycetaceae*	0.1 ± 0[bc]	0.5 ± 0.1[a]	0 ± 0[c]	0.2 ± 0.1[b]
		uncl. *Planctomycetaceae*	0 ± 0[b]	0.5 ± 0[a]	0.1 ± 0[b]	0.7 ± 0.2[a]
		uncl. *Planctomycetaceae*	0.1 ± 0[b]	0.9 ± 0.1[a]	0 ± 0[b]	0.1 ± 0[b]
		uncl. *Planctomycetaceae*	0 ± 0[b]	0 ± 0[b]	0.5 ± 0.1[a]	0 ± 0[b]
	Proteobacteria	uncl. *Devosia*	0.3 ± 0.1[b]	0.4 ± 0[b]	1 ± 0.2[a]	0.9 ± 0.2[a]
		uncl. *Rhizobiales*	0 ± 0[b]	0.6 ± 0.1[a]	0 ± 0[b]	0 ± 0[b]
		uncl. *Rhizobiales*	0.2 ± 0.1[b]	0.6 ± 0.1[a]	0.1 ± 0[b]	0 ± 0[b]
		uncl. *Rhizobiales*	0.5 ± 0.3[a]	0.7 ± 0.1[a]	0 ± 0[b]	0 ± 0[b]
		uncl. *Rhodospirillaceae*	0.4 ± 0.2[ab]	0.5 ± 0.2[a]	0.2 ± 0.1[ab]	0.1 ± 0[b]
		uncl. *Rhodospirillaceae*	1.9 ± 1.4[b]	1.4 ± 0.3[b]	4.4 ± 1.3[a]	1.5 ± 0.3[b]
		uncl. *Sphingomonadaceae*	0.1 ± 0.1[b]	0.5 ± 0.1[a]	0 ± 0[b]	0 ± 0[b]
		uncl. *Rhodocyclaceae*	0 ± 0[b]	0 ± 0[b]	1.6 ± 0.3[a]	0 ± 0[b]
		uncl. *Betaproteobacteria*	0 ± 0[b]	0.9 ± 0.2[a]	0 ± 0[b]	0 ± 0[b]
		uncl. *Betaproteobacteria*	0 ± 0[b]	0 ± 0[b]	0.6 ± 0.4[a]	0 ± 0[b]
		uncl. *Betaproteobacteria*	0 ± 0[b]	1.4 ± 0.2[a]	0 ± 0[b]	0.2 ± 0.1[b]
		uncl. *Betaproteobacteria*	0 ± 0b[c]	0.5 ± 0[a]	0 ± 0[c]	0.1 ± 0[b]
		uncl. *Betaproteobacteria*	0 ± 0[b]	0.8 ± 0.1[a]	0 ± 0[b]	0 ± 0[b]
		uncl. *Betaproteobacteria*	0.1 ± 0[b]	0.6 ± 0.1[a]	0 ± 0[b]	0 ± 0[b]
		uncl. *Desulfuromonadales*	0 ± 0[b]	0.6 ± 0.1[a]	0 ± 0[b]	0 ± 0[b]
		uncl. *Myxococcales*	0 ± 0[b]	0 ± 0[b]	0.3 ± 0.1[b]	1.3 ± 0.2[a]
		uncl. *Myxococcales*	0 ± 0[b]	0.5 ± 0.1[a]	0 ± 0[b]	0 ± 0[b]
		uncl. *Syntrophobacter*	0.1 ± 0[b]	0.7 ± 0.1[a]	0 ± 0[b]	0 ± 0[b]

Domain	Phylum	Last identified phylogenetic level	MS flooded	MS non-flooded	RS flooded	RS non-flooded
Bacteria		uncl. *Proteobacteria*	1 ± 0.7[b]	4.4 ± 0.8[a]	0.2 ± 0.1[b]	0.3 ± 0.1[b]
	uncl.	uncl.	0 ± 0[b]	0 ± 0[b]	0 ± 0[b]	3.1 ± 0.7[a]
		uncl.	0 ± 0[b]	0.6 ± 0.2[a]	0 ± 0[b]	0 ± 0[b]
		uncl.	0 ± 0[b]	0.6 ± 0.1[a]	0 ± 0[b]	0 ± 0[b]
		uncl.	0 ± 0[b]	0 ± 0[b]	0 ± 0[b]	0.6 ± 0.2[a]
		uncl.	0 ± 0[b]	0.6 ± 0.2[a]	0 ± 0[b]	0 ± 0[b]
		uncl.	0 ± 0[b]	0 ± 0[b]	0.7 ± 0.1[a]	0.1 ± 0[b]
		uncl.	0 ± 0[b]	1.5 ± 0.1[a]	0 ± 0[b]	0 ± 0[b]
		uncl.	0 ± 0[b]	0.6 ± 0[a]	0 ± 0[b]	0 ± 0[b]
		uncl.	0 ± 0[b]	0.7 ± 0.1[a]	0 ± 0[b]	0 ± 0[b]
		uncl.	0.1 ± 0.1[b]	0.2 ± 0.1[b]	0.1 ± 0.1[b]	0.8 ± 0.2[a]
		uncl.	0 ± 0[b]	1 ± 0[a]	0 ± 0[b]	0.1 ± 0[b]
		uncl.	0.2 ± 0.1[b]	0.8 ± 0.1[a]	0 ± 0[b]	0 ± 0[b]
		uncl.	0.5 ± 0.5[a]	0 ± 0[b]	0 ± 0[b]	0 ± 0[b]
		uncl.	1 ± 0.5[a]	0 ± 0[b]	0.3 ± 0.2[ab]	0 ± 0[b]
Eukarya	*Ascomycota*	uncl. *Sporormiaceae*	0 ± 0[b]	0 ± 0[b]	0.5 ± 0.1[a]	0.2 ± 0.1[b]
		uncl. *Trichocomaceae*	0.4 ± 0.1[a]	0.1 ± 0.2[ab]	0.2 ± 0.1[ab]	0 ± 0.1[b]
		uncl. *Helotiales*	0 ± 0[b]	0.4 ± 0.3[a]	0 ± 0[b]	0 ± 0[b]
		Chaetosphaeria vermicularioides	0 ± 0.1[b]	0.4 ± 0.1[a]	0 ± 0[b]	0 ± 0[b]
		uncl. *Nectriaceae*	0 ± 0[b]	0.4 ± 0.2[a]	0 ± 0[b]	0.1 ± 0.1[b]
		uncl. *Hypocreales*	0.1 ± 0[b]	1.8 ± 0.8[a]	0.4 ± 0.2[b]	0.3 ± 0.2[b]
		uncl. *Sordariales*	0.1 ± 0.2[b]	0.7 ± 0.2[a]	0 ± 0[b]	0 ± 0[b]
		uncl. *Microdochium*	0 ± 0[b]	0.5 ± 0.3[a]	0 ± 0[b]	0.2 ± 0.2[b]
	Basidiomycota	*Coniophora puteana*	0.2 ± 0.1[b]	0 ± 0[b]	1 ± 0.4[a]	0 ± 0[b]
		Cryptococcus terreus	0.3 ± 0.1[b]	5.7 ± 1.6[a]	0.1 ± 0[b]	0 ± 0[b]
		Cryptococcus terricola	0 ± 0[b]	5.1 ± 2[a]	0.1 ± 0.1[b]	0.2 ± 0.2[b]
		uncl. *Tremellales* fam. incertae sedis	0 ± 0[b]	0.5 ± 0.3[a]	0 ± 0[b]	0 ± 0[b]
	Chytridiomycota	uncl. *Spizellomyces*	0.1 ± 0.1[b]	0 ± 0[b]	1.4 ± 0.2[a]	0 ± 0[b]
		uncl. *Chytridiomycetes*	0 ± 0[b]	0 ± 0[b]	1.8 ± 0.5[a]	0 ± 0[b]
	Zygomycota	*Mortierella chlamydospora*	0.2 ± 0[b]	11.1 ± 4.3[a]	0.1 ± 0[b]	0 ± 0[b]
		uncl. *Mortierellales*	0.1 ± 0[b]	20.5 ± 6.2[a]	0.5 ± 0.2[b]	12.9 ± 9.0[ab]
		Umbelopsis ramanniana	0 ± 0[b]	1.1 ± 0.4[a]	0 ± 0[b]	0 ± 0[b]
	uncl.	uncl.	0 ± 0[b]	0.6 ± 0.2[a]	0 ± 0[b]	0.2 ± 0.2[b]

Domain	Phylum	Last identified phylogenetic level	MS flooded	MS non-flooded	RS flooded	RS non-flooded
Eukarya		uncl.	0 ± 0[b]	0 ± 0[b]	0.4 ± 0.1[a]	0 ± 0[b]
		uncl.	0.1 ± 0.1[b]	0.7 ± 0.5[a]	0 ± 0[b]	0.1 ± 0[b]
		uncl.	0 ± 0[b]	1.2 ± 0.7[a]	0 ± 0[b]	0 ± 0[b]
		uncl.	0 ± 0.1[b]	0 ± 0[b]	0.9 ± 0.4[a]	0 ± 0[b]
		uncl.	0 ± 0[b]	0.6 ± 0.4[a]	0 ± 0[b]	0.2 ± 0.1[b]
		uncl.	0 ± 0[b]	0 ± 0[b]	0.8 ± 0.5[a]	0 ± 0[b]
		uncl.	0 ± 0[b]	0 ± 0[b]	0.3 ± 0.2[a]	0 ± 0[b]
		uncl.	0 ± 0.1[b]	1 ± 0.3[a]	0 ± 0[b]	0 ± 0[b]
		uncl.	0.1 ± 0.1[b]	0.1 ± 0[b]	2.6 ± 1.9[a]	0 ± 0[b]
		uncl.	0.7 ± 0.1[a]	0 ± 0[c]	0.3 ± 0.1[b]	0 ± 0[c]
		uncl.	0 ± 0[b]	0 ± 0[b]	1.7 ± 1.4[a]	0 ± 0[b]
		uncl.	0.1 ± 0[b]	0.4 ± 0.1[b]	0.1 ± 0.1[a]	0 ± 0[b]
		uncl.	0.1 ± 0.1[b]	0 ± 0[b]	0.4 ± 0.1[a]	0 ± 0[b]
		uncl.	2.2 ± 1.1[a]	0.2 ± 0.1[b]	0.3 ± 0.1[b]	0.4 ± 0.2[b]
		uncl.	14.5 ± 9.7[a]	0 ± 0[b]	0.4 ± 0.2[b]	0 ± 0[b]
		uncl.	0 ± 0[b]	1.6 ± 0.6[a]	0 ± 0[b]	0.3 ± 0.3[b]
		uncl.	12.5 ± 8.1[a]	0 ± 0[b]	3.3 ± 4[ab]	0 ± 0[b]
		uncl.	0 ± 0[b]	0.9 ± 0.2[a]	0 ± 0[b]	0 ± 0[b]
